# The relationship between learning preferences (styles and approaches) and learning outcomes among pre-clinical undergraduate medical students

**DOI:** 10.1186/s12909-015-0327-0

**Published:** 2015-03-11

**Authors:** Siaw-Cheok Liew, Jagmohni Sidhu, Ankur Barua

**Affiliations:** 1 Department of Clinical Skills and Simulation Centre, International Medical University, No. 126, Jalan Jalil Perkasa 19, Bukit Jalil, 57000 Kuala Lumpur, Malaysia; 2 Department of Community Medicine, International Medical University, Kuala Lumpur, Malaysia

**Keywords:** Learning, Styles, Approach, Assist, Vark, Medical, Students

## Abstract

**Background:**

Learning styles and approaches of individual undergraduate medical students vary considerably and as a consequence, their learning needs also differ from one student to another. This study was conducted to identify different learning styles and approaches of pre-clinical, undergraduate medical students and also to determine the relationships of learning preferences with performances in the summative examinations.

**Methods:**

A cross-sectional study was conducted among randomly selected 419 pre-clinical, undergraduate medical students of the International Medical University (IMU) in Kuala Lumpur. The number of students from Year 2 was 217 while that from Year 3 was 202. The Visual, Auditory, Read/Write, Kinesthetic (VARK) and the Approaches and Study Skills Inventory for Students (ASSIST) questionnaires were used for data collection.

**Results:**

This study revealed that 343 students (81.9%) had unimodal learning style, while the remaining 76 (18.1%) used a multimodal learning style. Among the unimodal learners, a majority (30.1%) were of Kinesthetic (K) type. Among the middle and high achievers in summative examinations, a majority had unimodal (Kinaesthetic) learning style (30.5%) and were also strategic/deep learners (79.4%). However, the learning styles and approaches did not contribute significantly towards the learning outcomes in summative examinations.

**Conclusions:**

A majority of the students in this study had Unimodal (Kinesthetic) learning style. The learning preferences (styles and approaches) did not contribute significantly to the learning outcomes. Future work to re-assess the viability of these learning preferences (styles and approaches) after the incorporation of teaching-learning instructions tailored specifically to the students will be beneficial to help medical teachers in facilitating students to become more capable learners.

## Background

The learning styles and learning approaches constitute the learning preferences of undergraduate medical students. The “learning styles” are preferred methods of learning adopted by students in attaining, analysing and interpreting their knowledge [1]. The Visual, Auditory, Read/Write, Kinesthetic (VARK) model, developed by Fleming and Mills [2] is an acronym for Visual (V), Auditory (A), Read/Write (R) and Kinaesthetic (K) modalities which are used to assess learning styles.

The Visual (V) learner learns best by visualizing the information e.g., use of charts, diagram and mindmaps. The Auditory (A) learner learns best by hearing the information. The Read/Write (R) learner learns best when the information is displayed in words. The Kinaesthetic (K) learner learns best with practice or simulation [2].

The Approaches and Study Skills Inventory for Students (ASSIST) questionnaire which was developed by Entwistle and Ramsden [3] helps in the identification of students’ preferences in adopting any of the deep, surface or strategic approaches for learning. In the deep approach, the students actively relate their own ideas to the learning principles [4], use evidence and examine its logic and continue to monitor their own level of understanding [5].

In the strategic approach, students aim to achieve the highest scores possible. This involves good time management and study organization. Hence, they pay more attention to the content as well as assessment requirements [5]. In surface approach, learning of the students is restricted to routine memorisation as their intention is merely to complete the task [5]. The deep/strategic approach has been reported to be associated with better academic outcomes as compared to those with the surface approach [6].

Assessment is an important determinant which affects the way students learn and subsequently determines the learning outcomes [7]. It is a goal oriented approach which drives learning. Harris and Bell, [8] reported that students often modify their learning approaches in order to cope with the demands of assessment [8]. Many studies were conducted in the past to determine the association between learning approaches and summative examination results as a measure of learning outcomes. A majority of them had reported contradictory results on the relationship between learning approaches and outcomes [9-11]. The learning styles (VARK) were found to have a significant impact on the academic performances of undergraduate students of physiotherapy, nursing, dentistry and allied healthcare professional programmes [12-15].

The “meshing hypothesis” states that the learning outcomes could be highly achieved if learning was matched with predominant learning style of the learner [16,17]. However, Pashler et al. argue that the meshing theory was not necessarily correct because the undergraduate medical students were not always exposed to multiple types of learning styles for the same subject. Different subjects required different kinds of learning styles and instructions to optimally potentiate and benefit the students [17].

In the past, study methods were limited to lectures, tutorials and self-study. Major part of knowledge acquisition depended heavily on lectures. A shift to the skill of self-acquiring knowledge via information technology had widely changed the preferences and adaptation modes of knowledge acquirements of our 21^st^ century learners. In Asia, students are not spared from the advancement of technology, therefore their learning preferences had also changed with time along with this advancement. Many Asian higher institutions already have adopted information technology as one of their platforms for student learning and assessments [18,19]. With a variety of learning aids available these days, there is need to identify the most efficient way to match and deliver the teaching and learning instructions to help the undergraduate medical students to become more capable learners.

An in-depth understanding of modes of approach to learning would be beneficial for medical teachers to improve their delivery of learning materials. It would also help in tailoring the classroom instructions to suit the needs of undergraduate medical students in a more efficient and cost-effective manner. A majority of the previous studies on learning styles and learning approaches were conducted in western countries. This is the first study undertaken in a Malaysian context that examines the relationship between the learning preferences of pre-clinical undergraduate medical students and learning outcomes by using the VARK and ASSIST questionnaires. This study was conducted to investigate the learning preferences (styles and approaches) of the pre-clinical (Year 2 & 3) undergraduate medical students and also to associate the learning preferences (styles and approaches) with learning outcomes as performances in the summative examinations.

## Methods

This cross-sectional study was conducted for four months (September 2013 to December 2013) at the Clinical Skills and Simulation Centre, International Medical University, Kuala Lumpur, Malaysia.

### Sample size

The sample frame consisted of 470 undergraduate medical students. Among these, 230 belonged to Year 2 while 240 belonged to Year 3 of the undergraduate medical programme. The probability sample size for finite population was calculated by using confidence interval = 95%, absolute precision of estimate = 5% and prevalence rate of any one of the learning styles among the students = 50% (as previously no similar study was conducted in Malaysia). The minimum sample size required was estimated to be 293 which included 145 from Year 2 and 148 from Year 3 respectively. However, depending on feasibility and applying the inclusion and exclusion criteria, the total number of respondents in this study was found to be 419.

### Inclusion and exclusion criteria

All the pre-clinical, undergraduate medical students of Year 3, who attended their summative first Professional Examination were invited to participate in this study. All the undergraduate medical students of Year 2, who attended their summative examination at end of second semester, were also invited to participate in this study. These summative examinations were written/theoretical papers covering various aspects of basic sciences i.e. physiology, anatomy, biochemistry, pharmacology and pathology. All these examinations were conducted in English. Participation in this study was on a voluntary basis. All the Year 2 and Year 3 students who provided informed written consent to participate in this study were included. The designated undergraduate medical students who were absent and could not be contacted during the data collection period were excluded from this study. Stratified random sampling method was adopted to randomly select the respondents from both these strata of year 2 and Year 3. The required sample was redistributed according to the proportion of sampling frame in these two groups.

### Study instruments

The demographic data, educational background and preferred methods of studying among the undergraduate medical students were captured in a pre-tested pro forma. The latest version (v7.1) of VARK (Visual/Aural/ReadWrite/Kinesthetic) was used to assess the learning styles. The Cronbach’s Alpha score for individual components of VARK were found to be V (0.85), A (0.82), R (0.84), K (0.77) respectively. The VARK questionnaire consisted of 16 questions with four options each and the respondents could select more than one response for each question if deemed suitable [20]. A short version of ASSIST (Approaches and Study Skills Inventory for Students) was used to assess the learning approaches. The Cronbach’s Alpha score for individual components of ASSIST were as follows: deep (0.85), strategic (0.88) and surface (0.81). The ASSIST questionnaire required the respondents to rate the degree of their agreement on a five-point Likert-scale (ranging from strongly disagree to strongly agree) with a series of related items that covered various aspects of a specific construct [21]. The students were provided 30 minutes to complete these questionnaires.

### Ethical considerations

The study protocol was in compliance with the Helsinki Declaration and it was approved by the Joint Committee of Research and Ethics of the International Medical University (IMU) in Kuala Lumpur, Malaysia. Informed written consent was obtained from every participant before data collection and confidentiality of all responses was maintained throughout this study.

### Data collection procedure

After obtaining informed written consent, the participants were invited to respond to the questionnaires anonymously. Three consecutive attempts were made on separate occasions to contact a designated student who was found to be absent on the first day of data collection. If a student was found to be absent on all four occasions then he/she was excluded from this study and replaced by another randomly selected respondent belonging to the same group. The completed VARK questionnaire was evaluated using the scoring instructions provided in the guidelines. The completed ASSIST questionnaire was evaluated by adding the responses across all the items which produced a total scale score which identified whether the students adopted deep, strategic or surface approach during learning. The summative examination scores of the students of Year 3 (first professional examination) and Year 2 (end of second semester examination) were compared to the VARK scores, ASSIST scores and the number of attendances at medical lectures. The educational background of the students was compared against their summative examination scores along with VARK and ASSIST scores.

### Statistical analysis

The data collected were tabulated and analysed by using the Statistical Package for Social Sciences (SPSS) version 17.0. Results were presented in terms of number and proportions. The Chi-square Test and Odds Ratio along with its 95% Confidence Interval (CI) were used for comparison purposes. In this study, *p*-value <0.05 was considered as statistically significant.

## Results

### Baseline information

Table 1 shows the socio-demographic information of the students (n = 419). The Year 2 students (n = 217) are those who had sat for their end of second semester summative examination while the Year 3 students (n = 202) are those who had taken their first professional summative examination at the end of Year 2. A majority of the students are females (55.1%). A majority of the students are Malaysians (91.4%) while the minority are international students (8.6%). The Malaysian students are those who had either attended the National educational system (n = 320) or those who have undergone the private/international schooling system in Malaysia (n = 63). The international students (n = 36) are from various countries like Australia, Nigeria, Singapore, Indonesia, Brunei, New Zealand, Canada, Hong Kong, Korea, Japan and Myanmar. The undergraduate students (n = 410) are those who are taking medical course as their first degree whereas postgraduate students (n = 9) are those who have previously obtained a previous graduate degree and this medical course is their additional graduate degree programme. Most of these students have GCSE A-Levels as their pre-university entrance qualification (n = 279), others have Foundation in Science and International Baccalaureate as their pre-university qualifications. The mid/high achievers are those who scored 65% and above at their summative examinations^a^.

**Table 1:** **Baseline demographic data**

Components	Year 2	Year 3	Total
	n (%)	n (%)	n (%)
No of students	217 (51.8)	202 (48.2)	419 (100)
Malaysian students	196 (90.3)	187 (92.6)	383 (91.4)
International Students	21 (9.7)	15 (7.4)	36 (8.6)
Undergraduate	211 (97.2)	199 (98.5)	410 (97.9)
Postgraduate	6 (2.8)	3 (1.5)	9 (2.1)
Male	98 (45.2)	90 (44.6)	188 (45)
Female	119 (54.8)	112 (55.4)	231 (55)
Age (mean ± SD)	20.64 ± 1.24	21.57 ± 1.12	

#### Comparison of learning approaches

Table 2 shows the comparison of the learning approaches. A majority of the Year 2 (74.7%) and Year 3 (80.2%) students were found to be deep/strategic learners. There was no significant difference between the learning approaches of the younger medical students as compared to their immediate senior counterparts (p = 0.176). Similar to the international students (86.1%), a majority of the Malaysian students were found to be deep/strategic learners (76.5%). The deep/strategic groups were found to use all the four aids to learning (online lectures 78.2%, books 77.8%, discussion 75.8% and own notes 84.8%) than their counterpart. Most of the deep/strategic learners read textbooks (80.2%) and listened to online lectures (73.1%). They were less engaged in discussion (22.2%) and did not have their own notes for revision (20.7%). There was no significant gender variation in learning approaches (p = 0.207). The possession of a primary degree prior to joining medical school did not contribute significantly to the types of learning approaches among the respondents. Seniority in terms of age was not found to be significantly associated with the types of learning approaches. There was also no significant relationship between study approaches and study styles (p = 0.592). Though a majority of the mid/high achievers were deep/strategic learners, this association was not found to be statistically significant^b^.

**Table 2:** **Comparison of the learning approaches**

	Superficial	Deep/Strategic	Chi square	Odds ratio
	n (%)	n (%)	p-value	
**Year 2**	**55 (25.3)**	**162 (74.7)**	***0.176***	***1.375***
**Year 3**	**40 (19.8)**	**162 (80.2)**		
**(Yr2 & Yr3)**	**95 (22.7)**	**324 (77.3)**		
**Malaysian**	**90 (23.5)**	**293 (76.5)**	***0.188***	***1.904***
**International**	**5 (13.9)**	**31 (86.1)**		
**Online Lectures**	**66 (21.8)**	**237 (78.2)**	***0.482***	***1.197***
**Books**	**74 (22.2)**	**260 (77.8)**	***0.616***	***1.153***
**Discussion**	**23 (24.2)**	**72 (75.8)**	***0.684***	***0.894***
**Own Notes**	**12 (15.2)**	**67 (84.8)**	***0.078***	***1.803***
**Male**	**48 (25.5)**	**140 (74.5)**	***0.207***	***1.342***
**Female**	**47 (20.3)**	**184 (79.7)**		
**Undergraduate**	**92 (22.4)**	**318 (77.6)**	***0.440***	***0.579***
**Postgraduate**	**3 (33.3)**	**6 (66.7)**		
**Age ≤20**	**27 (20.9)**	**102 (79.1)**	***0.570***	***0.864***
**Age > 20**	**68 (23.4)**	**222 (76.6)**		
**Unimodal**	**76 (22.2)**	**267 (77.8)**	***0.592***	***0.854***
**Multimodal**	**19 (25)**	**57 (75)**		
**Mid/High Achievers**	**66 (20.6)**	**255 (79.4)**	***0.062***	***1.624***
**Low Achievers**	**29 (29.6)**	**69 (70.4)**		

p-value < 0.05 was considered as statistically significant.

#### Comparison of learning styles

Table 3 shows the distribution of learning styles. In this study, 343 respondents (81.9%) had adopted unimodal learning style while the remaining 76 students (18.1%) embraced multimodal learning styles. However, there was no significant difference between the two groups (p = 0.592). The learning styles between the Year 2 and Year 3 undergraduate medical students were also not significantly different (p = 0.243). A majority of the students who embraced unimodal learning style were found to be Kinesthetic (K) learners (30.1%). The Malaysian (29.8%) and the international students (33.3%) mostly adopted the Kinesthetic (K) learning style. There was no significant gender variation in learning styles as equal proportions of males (31.9%) and females (28.6%) had adopted Kinesthetic (K) learning style. Those who did prior graduation before joining the medical course (33.3%) preferred the Read/Write (R) learning styles. Both the age groups (those above and below 20 years) preferred the Kinesthetic (K) learning styles. However, a higher proportion of those aged 20 years and above adopted multimodal learning styles (21%) as compared to those aged less than 20 years (11.5%). Within the Kinaesthetic (K) group, the more preferred methods of studying were online lectures (65.9%) and textbooks (79.4%). A majority of the mid/high (30.5%) and low achievers (28.6%) had Kinesthetic (K) learning styles. Although not statistically significant (p = 0.714), a majority of the unimodal learners were mid/high achievers (82.2%) as compared to the multimodal learners.

**Table 3:** **Comparison of the study styles**

	V	A	R	K	Chi square
	n (%)	n (%)	n (%)	n (%)	p-value
**Year 2**	**23 (10.6)**	**46 (21.2)**	**47 (21.7)**	**69 (31.8)**	**0.243**
**Year 3**	**24 (11.9)**	**31 (15.3)**	**46 (22.8)**	**57 (28.2)**	
**Overall (Yr2 & Yr3)**	**47 (11.2)**	**77 (18.4)**	**93 (22.2)**	**126 (30.1)**	
**Malaysian**	**42 (11)**	**72 (18.8)**	**86 (22.5)**	**114 (29.8)**	**0.911**
**International**	**5 (13.9)**	**5 (13.9)**	**7 (19.4)**	**12 (33.3)**	
**Male**	**23 (12.2)**	**36 (19.1)**	**43 (22.9)**	**60 (31.9)**	**0.358**
**Female**	**24 (10.4)**	**41 (17.7)**	**50 (21.6)**	**66 (28.6)**	
**U’grade**	**45 (11)**	**77 (18.8)**	**90 (22)**	**124 (30.2)**	**0.482**
**P’grade**	**2 (22.2)**	**0 (0)**	**3 (33.3)**	**2 (22.2)**	
**≤20 years**	**14 (10.9)**	**29 (22.5)**	**26 (20.2)**	**45 (34.9)**	**0.096**
**>20 years**	**33 (11.4)**	**48 (16.6)**	**67 (23.1)**	**81 (27.9)**	
**Low achievers**	**12 (12.2)**	**23 (23.5)**	**16 (16.3)**	**28 (28.6)**	**0.390**
**Mid/High achievers**	**35 (10.9)**	**54 (16.8)**	**77 (24)**	**98 (30.5)**	
**Deep/Strategic**	**42 (13.0)**	**55 (17.0)**	**71 (21.9)**	**99 (30.6)**	**0.214**
**Superficial**	**5 (5.3)**	**22 (23.2)**	**22 (23.2)**	**27 (28.4)**	

p-value < 0.05 was considered as statistically significant.

## Discussion

It was observed that most of the respondents in this study were Kinesthetic learners (30.1%) regardless of their gender, age, nationality and educational backgrounds. This finding was similar to those reported by Kharb et al. for Indian medical students [1] as well as Baykan and Nacar for Turkish medical students [22].

Lujan and DiCarlo reported that the most preferred learning style of first year medical students from Indiana, USA was of Read/Write (R) modality [23]. In this study, the same was observed amongst the medical students who already possessed a primary degree prior to their entry to the medical school (postgraduate students). However, Nuzhat et al. reported that the most preferred learning style among medical students in Saudi Arabia was the auditory mode [24]. The dental students in Philadelphia were found to prefer the Visual (V) learning more than the Kinesthethic (K) learning [25]. These variance in learning styles according to countries could be due to cultural differences as well as previous exposures to different teaching styles during the years at pre-medical schools.

A majority of the participants in this study had unimodal learning style (81.9%). A study by Fleming on 31,243 students reported that the ratio of unimodal against multimodal learning style preference was 42:58 [20,25]. Other studies reported the preferences for multimodal learning styles with percentages ranging between 53% and 85%. The proportions of students preferring multimodal learning style from various studies were as follows: proportion reported by Dinakar et al. was 58% [26], Lujan et al. was 63.8% [23], Baykan and Nacar was 63.9% [22], Nuzhat et al. was 72.6% [24], Bahadori et al. was 59% [27] and Ding et al. was 85.7% [28]. However, in this study, only a small proportion of Malaysian students adopted the multimodality learning style. This could be due to their exposure to different kinds of teaching learning instructions at pre-medical schools. In Malaysia, the educational system is mostly didactic in nature, with very minimal hands-on, discussion and practical sessions. However, a majority of the respondents with multimodal learning styles belonged to the age group of (20-29) years as they were graduates before joining the medical programme. This finding was similar to a study conducted by McKean J et al. during 2009 in Hong Kong [29].

There was no significant difference in learning styles and performances at the summative examinations. This could be due to the fact that learning styles mainly focussed on strategies adopted by students in acquiring knowledge. There was no evidence in this study that any particular learning style in itself was superior as compared to others in the attainment of academic success. However, there were some reports that the knowledge and understanding of one’s own learning style could greatly enhance one’s success in the summative examinations [30,31]. The students in this study were not provided with any specific teaching and learning method which was tailored according to their preferred learning style. A blend of activities covering all the learning modalities were made available to them. The response of the participants would have been different if they were exposed to matched learning strategies according to their respective learning styles.

In this study, a majority of the students among the mid/high achievers category (79.4%) embraced the deep/strategic learning approach. Interestingly, a majority of the low achievers were also found to be deep/strategic learners. Hence, this difference was not found to be statistically significant. However, some of the previous studies reported that students with deep/strategic learning approaches performed better during their final summative examinations as compared to their superficial learning counterparts [32-34]. We concur with a study by Newble et al. which found that undergraduate medical students with deep/strategic learning approach did not perform better during summative examinations [35].

The deep/strategic learning approach was believed to be able to help students to attain more successes in summative examinations as compared to the superficial learning approach [6]. Although there was no statistically significant relationship between the learning approaches (deep/strategic) and learning outcomes in terms of summative examinations in this study, many other studies had reported a positive beneficial association [9-11].

Both the VARK and ASSIST questionnaires analysed only one aspect of the learning preferences (styles and approaches). Hence, multiple aspects of learning preferences (styles and approaches) could not be assessed in this study. The students in this study were not exposed to their preferred learning styles and approaches during their learning activities prior to the summative examinations. This prevented the investigators from studying the true effects of their learning preferences on learning outcomes.

The teaching and learning strategies should be redesigned to promote deep/strategic learning among the pre-clinical undergraduate medical students. The teaching and learning instructions should be tailored according to the learning preferences (styles and approaches) of the students. More active hands-on learning strategies like simulations, role playing, problem based discussions and debates should to be incorporated in the teaching and learning activities. This would create better learning environment for the kinesthetic learners.

## Conclusions

This study revealed that the learning preferences (styles and approaches) of the students in this study did not contribute significantly towards their learning outcomes. Tailoring the delivery of teaching and learning instructions matching with the learning preferences (styles and approaches) of the pre-clinical undergraduate medical students followed by a re-assessment of their performances at summative examinations would be beneficial to genuinely gauge the potential of these teaching-learning strategies. This study should be replicated in other medical institutions in this region to confirm the findings.

### Endnotes

^a^At the International Medical University, a score of 65% determines the cut off point for those who scores a grade B and above- therefore would be defined as mid/high achievers compared to those who scored lower than this.

^b^We have combined the deep/strategic approach instead of having them as a separate entity because a published data have shown that the “deep, strategic approach, without any elements of surface apathetic, is generally associated with successful academic performance” (Entwistle, [6]).
